# Oxidative stress caused by activation of NADPH oxidase 4 promotes contrast-induced acute kidney injury

**DOI:** 10.1371/journal.pone.0191034

**Published:** 2018-01-12

**Authors:** Bo Young Jeong, Hoi Young Lee, Chang Gyo Park, Jaeku Kang, Seong-Lan Yu, Du-ri Choi, Seung-Yun Han, Moon Hyang Park, Sungkwon Cho, Soo Young Lee, Won-Min Hwang, Sung-Ro Yun, Hye-Myung Ryu, Eun-Joo Oh, Sun-Hee Park, Yong-Lim Kim, Se-Hee Yoon

**Affiliations:** 1 Department of Pharmacology, College of Medicine, Konyang University, Daejeon, Republic of Korea; 2 Myunggok Medical Research Institute, College of Medicine, Konyang University, Daejeon, Republic of Korea; 3 Department of Anatomy, College of Medicine, Konyang University, Daejeon, Republic of Korea; 4 Department of Pathology, College of Medicine, Konyang University, Daejeon, Republic of Korea; 5 Division of Nephrology and Department of Internal Medicine, Myunggok Medical Research Institute, College of Medicine, Konyang University, Daejeon, Republic of Korea; 6 Division of Nephrology and Department of Internal Medicine, Kyungpook National University Hospital, Daegu, Republic of Korea; National Institutes of Health, UNITED STATES

## Abstract

Contrast-induced acute kidney injury (CIAKI) is a leading cause of acute kidney injury following radiographic procedures. Intrarenal oxidative stress plays a critical role in CIAKI. Nicotinamide adenine dinucleotide 3-phosphate (NADPH) oxidases (Noxs) are important sources of reactive oxygen species (ROS). Among the various types of Noxs, Nox4 is expressed predominantly in the kidney in rodents. Here, we evaluated the role of Nox4 and benefit of Nox4 inhibition on CIAKI using *in vivo* and *in vitro* models. HK-2 cells were treated with iohexol, with or without Nox4 knockdown, or the most specific Nox1/4 inhibitor (GKT137831). Effects of Nox4 inhibition on CIAKI mice were examined. Expression of Nox4 in HK-2 cells was significantly increased following iohexol exposure. Silencing of Nox4 rescued the production of ROS, downregulated pro-inflammatory markers (particularly phospho-p38) implicated in CIAKI, and reduced Bax and caspase 3/7 activity, which resulted in increased cellular survival in iohexol-treated HK-2 cells. Pretreatment with GKT137831 replicated these effects by decreasing levels of phospho-p38. In a CIAKI mouse model, even though the improvement of plasma blood urea nitrogen was unclear, pretreatment with GKT137831 resulted in preserved structure, reduced expression of 8-hydroxy-2’-deoxyguanosine (8OHdG) and kidney injury molecule-1 (KIM-1), and reduced number of TUNEL (terminal deoxynucleotidyl transferase dUTP nick end labeling)-positive cells. These results suggest Nox4 as a key source of reactive oxygen species responsible for CIAKI and provide a novel potential option for prevention of CIAKI.

## Introduction

Iodinated contrast medium (CM)-induced acute kidney injury (CIAKI) is an acute deterioration of renal function following administration of CM in the absence of any other known reason [1] CIAKI remains the leading cause of iatrogenic acute kidney injury (AKI) following radiographic procedures. CIAKI has been reported to occur in 1–6% of hospitalized patients [2]. In particular, the incidence of CIAKI may reach as high as 50% in high-risk patients, such as those with dehydration, diabetic nephropathy, renal impairment, volume depletion, or congestive heart failure, and in elderly individuals [3, 4]. Additionally, administration of a high volume of CM, contrast osmolarity, and the concomitant use of medications have been indicated as risk factors for CIAKI [5, 6]. Despite the risk of CIAKI, the use of radiocontrast procedures for computerized tomography and vascular interventions, particularly in high-risk and elderly patients with major comorbidities, has continued to increase [1, 7, 8].

The exact mechanism of CIAKI is not fully understood. It has been suggested that CM increases osmotic load, decreases renal blood flow, and induces renal arterial constriction. Such a condition promotes generation of ROS and results in ischemic tubular injury, and can be a reason for direct tubular toxicity [1, 9]. Oxygen radicals are endogenously produced and different sources of ROS, ranging from xanthine-xanthine oxidase and mitochondria to nicotinamide adenine dinucleotide 3-phosphate (NADPH) oxidase (Nox) enzymes, have been identified [10]. The Nox family is composed of seven members (Nox1–5, DUOX1, and DUOX2). Among them, Nox4 is the predominant form in the kidney and has been implicated in the production of ROS in the kidneys in both basal and pathologic conditions such as diabetic nephropathy and chronic kidney disease [10–14]; upregulation of Nox4 may be important in renal oxidative stress and kidney injury. Although growing evidence indicates the involvement of Nox4 in renal pathology, few studies have evaluated the role of Nox4 in AKI [10, 11]. To determine the role of Nox4 in CIAKI, we used the most specific Nox1/4 inhibitor GKT137831 [2-(2-chlorophenyl)-4-[3-(dimentylamino)phenyl]-5methyl-1H-pyrazolo[4,3-c]pyridine-3,6(2H,5H)-dione] (S1 Fig). GKT137831 binds to the extracellular portion of the catalytic subunit of the Nox enzymes. As no effect on Nox4 or Nox1 protein expression was observed for GKT137831, it appears to target the enzymatic activity of Nox4 and Nox1 [15, 16]. Based on these observations, we investigated the role of Nox4 and the benefit of inhibition of Nox4 using in vivo and in vitro models of CIAKI.

## Materials and methods

### Reagents

Iohexol was purchased from Tokyo Chemical Industry (Chuo-ku, Tokyo, Japan). GKT137831, a Nox1/4 selective inhibitor, was kindly provided by Genkyotex (Chemin des Aulx, Plan-les-Ouates, Switzerland).

### Cell culture

HK-2 cells (human renal proximal tubular epithelial cells) were obtained from the Korean Cell Line Bank (KCLB, Seoul, South Korea). HK-2 cells were cultured in RPMI-1640 supplemented with 10% fetal bovine serum and 1% penicillin/streptomycin. Cells were incubated at 37°C under 5% CO_2_ in a humidified incubator. Subconfluent cells were starved for 12 h in RPMI without fetal bovine serum and then incubated with either control medium (serum-free RPMI) or contrast agents diluted in serum-free medium for the indicated times.

### Plasmid and siRNAs transfection

HK-2 cells were transiently transfected using Lipofectamine 3000 or RNAiMAX (Invitrogen, Carlsbad, CA, USA) according to the manufacturer’s instructions. Nox4 cDNA was subcloned into a pcDNA3 vector; the empty pcDNA3 vector was used as a negative control. Nox4 cDNA and pcDNA3 vector were kindly provided by the Bae laboratory (Ehwa Womans University, Korea). siRNAs targeting Nox4 were purchased from Dharmacon (Lafayette, CO, USA). Control scrambled-sequence siRNA was purchased from Invitrogen. The greatest efficiency was observed at a concentration of 50 pmol/ml, and thus all experiments were conducted at 50 pmol/ml (S2 Fig).

### Immunoblotting

HK-2 cells were washed with phosphate-buffered saline (PBS) and incubated for 15 min at room temperature in ice-cold radioimmunoprecipitation assay (RIPA) buffer. Tissue extracts were also prepared from whole mouse kidneys. Whole cell lysates and protein extracts (50 μg) were subjected to sodium dodecyl sulfate (SDS)-polyacrylamide gel electrophoresis on 8% gels and transferred to polyvinylidene difluoride (PVDF) membranes. Nonspecific sites were blocked with 5% nonfat dried milk for 2 h at room temperature, and membranes were then incubated with primary antibodies in PBS (pH 7.2) overnight at 4°C. Antibodies used for immunoblotting were as follows: anti-Nox4 (Abcam, Cambridge, MA, USA, #ab109225), anti-phospho-p38 (Cell Signaling Technology, Danvers, MA, USA, #4511S), anti-phospho-JNK (Cell Signaling Technology, #4668S), anti-p38 (Santa Cruz Biotechnology, Santa Cruz, CA, USA, #sc535), anti-JNK (Santa Cruz Biotechnology, #sc474), anti-phospho-ERK (Santa Cruz Biotechnology, #sc7383), anti-ERK (Santa Cruz Biotechnology, #sc154) anti Bax (Santa Cruz Biotechnology, #sc493), anti Bcl-2 (Santa Cruz Biotechnology, #sc7382), anti SOD2 (Abcam, #ab13533), anti GPx- 1/2 (Santa Cruz Biotechnology, #sc133160), and anti-β-actin (Santa Cruz Biotechnology, #sc47778). Anti-pERK (Cell Signaling Technology, #4370), anti ERK (Cell Signaling Technology, #9102), anti-phospho-p38 (Cell Signaling Technology, #9211), anti-p38 (Cell Signaling Technology, #9212), anti-phospho-JNK (Cell Signaling Technology, #9251), JNK (Cell Signaling Technology, #9252), and anti β-actin (Sigma Aldrich, #1978) were used for tissue western blotting. The membranes were washed twice with wash buffer (0.1% Tween 20 in PBS) and incubated with horseradish peroxidase-conjugated secondary antibodies for 2 h at room temperature. After washing twice with wash buffer, the bands were visualized using enhanced chemiluminescence (Thermo Fisher Scientific Inc., Rockford, IL, USA) using an Image Quant 400 instrument (GE Healthcare, Buckinghamshire, UK).

### Cell viability

Cell viability was measured using an ATPlite kit (Perkin Elmer-Cetus, CT, USA) and the 3-(4,5-dimethylthiazole-2-yl)-2,5-diphenyltetrazolium bromide (MTT) assay. For the ATP assay, the lysates were stabilized using mammalian cell lysis solution. After 5 min of incubation, substrate solution was added to each sample. The luminescence was measured using a Lumat LB953 luminometer (EG&G Berthhold, Bad Wildbad, Germany). For the MTT assay, subcultured cells (1 × 10^4^ cells/ml) were exposed to various concentrations of the test compounds in a 24-well plate and incubated for 72 h at 37°C in 5% CO_2_. Following the 72 h incubation, 5 mg/ml MTT solution (Sigma) was added to the wells and the cells were incubated for a further 4 h. The supernatant was then removed and 1 ml of dimethyl sulfoxide (DMSO) was added to each well. Immediately after purple formazan crystals formed and dissolved, the solution was collected and pipetted into a 96-well plate. The optical density was measured at 590 nm using the optical density at 630 nm as reference (VICTOR X3; PerkinElmer, Waltham, MA, USA).

### Caspase 3/7 activity assays

Cells seeded in 96-well plates were incubated with Caspase-Glo 3/7 substrate reagent (Promega, Madison, WI, USA) at 37°C for 30 min. The samples were transferred to white-walled plates, and the luminescence signal was measured using a Lumat LB953 luminometer (EG&G Berthhold).

### Quantitative real-time PCR

Briefly, total cellular RNA was extracted from a given cell line with TRIzol reagent (Invitrogen). Total cellular RNA (2 μg) was isolated from cultured cells and reverse-transcribed using oligo (dT) and M-MLV reverse transcriptase (Promega). The cDNA concentration was adjusted to 100 ng/ml, and PCR was performed using an iQ SYBR Green Supermix (Bio-Rad Laboratories, Hercules, CA, USA) with 5 min of predenaturation at 95°C, followed by 40 cycles of DNA amplification including annealing at 60°C for 30 s and extension at 72°C for 30 s. Assays used the following primer sets: NOX4, 5′-GGCTGGAGGCATTGGAGTAA-3′ (forward) and 5′-CCAGTCATCCAACAGGGTGTT-3′ (reverse); β-actin, 5′-TCAAGATCATTGCTCCTCCTG-3′ (forward) and 5′-CTGCTTGCTGATCCACATCTG-3′ (reverse). The relative expression of the reverse transcription (RT)-PCR products was determined using the ΔCt method. This method calculates the relative expression using the following equation: fold induction = 2 − (ΔCt), where Ct is the threshold cycle and ΔCt = (Ct_gene of interest_−Ct_β-actin_). Each sample was run in triplicate, and three independent experiments were performed. The mean Ct was used in the ΔCt equation.

### Amplex red assays

Hydrogen peroxide (H_2_O_2_, end product of Nox4) was measured with the Amplex red assay using the Amplex red hydrogen peroxide/peroxidase assay kit (Invitrogen). Reactions containing 50 μM Amplex Red reagent and 0.1 U/ml catalase in 50 mM sodium phosphate buffer (pH 7.4) were prepared in a darkroom under red light. White enzyme-linked immunosorbent assay (ELISA) plates containing the samples (100 μL/well) in triplicate were either kept in the dark for 30 or 60 min or under ambient laboratory light. Fluorescence (excitation: 535 nm, emission: 595 nm) was measured on an HTS Multi-Label Reader (Perkin Elmer).

### Intercellular ROS detection

The oxidative fluorescent dye DHE was obtained from Thermo Fisher Scientific Inc. Cells were incubated on glass slides in 12-well plates. After fixation with cold methanol for 10 min and permeabilization for 30 min with 1% bovine serum albumin, cellular immunofluorescence was evaluated by staining with DHE for 40 min. The nuclei were counterstained with 4′,6-diamidino-2-phenylindole (DAPI; Molecular Probes, Carlsbad, CA, USA), and the cells were then washed with ice-cold PBS three times for 5 min and examined by confocal microscopy (LSM710; Carl Zeiss, Jena, Germany). Representative images are shown from at least three independent experiments with similar results.

### Mice

Male C57BL/6 mice were purchased from Samtako (Korea). The Institutional Animal Care and Use Committee of Konyang University approved the animal care protocol for the experiments performed in the study.

### Murine model of CIAKI

CIAKI was induced in mice as described previously. After overnight (16 h) water deprivation and inhibition of prostaglandin and nitric oxide synthesis, mice were injected intraperitoneally with the low-osmolar monomeric iodinated radiocontrast medium iohexol (Omnipaque, 4,000 mg iodine/kg). For inhibition of cyclooxygenase and nitric oxide synthesis, mice were injected intraperitoneally with indomethacin (10 mg/kg dissolved in ethanol) and N-nitro-l-arginine methyl ester (L-NAME; 10 mg/kg dissolved in 0.9% saline), respectively, 15 min before iohexol injection; this model reliably produces nephropathy following radiocontrast injection and has been previously validated in mice and rats [17, 18]. Animals were then given access to food and water and euthanized 24 h later for serum BUN collection and kidney removal. Animals were sacrificed under anesthesia with ketamine/xylazine (0.5 ml of 100 mg/ml ketamine combined with 0.05 ml of 20 mg/ml xylazine) at a dosage of 0.55 ml/100 g body weight.

**Fig 1:**
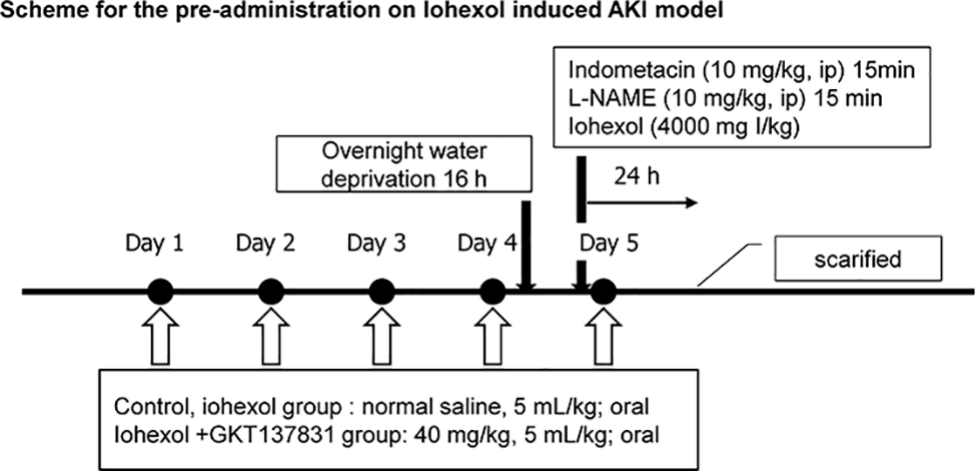
Experimental protocol for the iohexol-induced AKI model. GKT137831 (6 mg/ml or 5 ml/kg) or normal saline (5 ml/kg) was administered orally once a day during four days prior to the injection of iohexol and once after the injection of iohexol. The mice were injected with 4,000 mg/kg iohexol.

To determine whether GKT137831 protected against murine CIAKI, we assigned mice to the following treatment groups: 1) control group (CONT; n = 5), 2) CIAKI group (n = 8), and 3) GKT137831 treatment (GKT) + CIAKI group (n = 8). Mice in the GKT group received GKT137831 (40 mg/kg) once a day for 5 days prior to the CIAKI-inducing injections. After 24 h, both kidneys were harvested for associated measurements. The right kidney was fixed in 4% paraformaldehyde for histological assessments. Blood samples were collected for isolation of serum and stored at -80°C (Fig 1).

### Analysis of biochemical markers in blood

An automatic biochemical analyzer (Hitachi 7600; Ichige, Japan) was employed to determine blood urea nitrogen (BUN) and serum creatinine (SCr) levels. superoxide dismutase (SOD) activity was determined using the SOD assay kit following the manufacturer’s protocol, and SOD activity was expressed as μg/ml. Lipid peroxidation, as measured by malondialdehyde (MDA) levels, was determined in serum using an MDA assay kit (Sigma) following the manufacturer’s protocol. The serum MDA results were expressed as μM.

### Histologic examination and histopathology scoring

To detect injury of the kidney, the fixed left kidney was dehydrated in ethanol and embedded in paraffin. Kidney tissue blocks were cut into 2-μm-thick sections and subjected to hematoxylin-eosin (H&E) staining and periodic acid Schiff (PAS) staining. Histological scoring was assessed by grading tubular necrosis, loss of brush borders, cast formation, and tubular dilatation in 10 randomly chosen, nonoverlapping fields. The degree of renal injury was estimated based on the following criteria: 0, none; 1, 0–10%; 2, 11–25%; 3, 26–45%; 4, 46–75%.

### Immunohistochemistry

The paraffin samples were cut at 2-μm thickness, deparaffinized, and rehydrated. After inactivation of endogenous peroxidase with 3% H_2_O_2_, slides were pre-incubated with 10% fetal calf serum in Dako buffer (Dako) to block non-specific reactions. The samples were incubated with rabbit polyclonal anti TIM-1 (10 μg/ml, ThermoFisher, PA5-20244) and goat polyclonal antibody against 8-hydroxyguanosine (8-OHdG) (2 μg/ml, Abcam, San Francisco, CA, USA, ab10802) overnight at 4°C. Anti-Nox1 (Abcam, San Francisco, CA, USA, ab131088), anti Nox2/gp91phox (Abcam, San Francisco, CA, USA, ab80508), and anti-Nox4 (Abcam, San Francisco, CA, USA, ab133302) were used to detect the various NDAPH homologues. Secondary antibodies were applied for 1 h at room temperature in 10% FCS/Dako buffer. Finally, the sections were incubated with avidin peroxidase (1:100, Sigma Aldrich) in 10% FCS/Dako buffer for 1 h at room temperature. Antibody binding was routinely visualized using DAB (Sigma Aldrich). The sections were counterstained with haematoxylin, dehydrated, and mounted in Eukitt.

### Terminal deoxynucleotidyl transferase dUTP nick end labeling (TUNEL) assay

The TUNEL assay was conducted using a TUNEL detection kit according to the manufacturer’s instructions (HRP kit DBA; Apotag, Milan, Italy). Briefly, sections were incubated with 15 μg/ml proteinase K for 15 min at room temperature and then washed with PBS. Endogenous peroxidase was inactivated with 3% H_2_O_2_ for 5 min at room temperature and then washed with PBS. Sections were immersed in terminal deoxynucleotidyltransferase and biotinylated dUTP in TdT buffer, incubated in a humid atmosphere at 37°C for 90 min, and then washed with PBS. The sections were incubated at room temperature for 30 min with anti-horseradish peroxidase-conjugated antibodies, and the signals were visualized with diaminobenzidine. The number of TUNEL-positive cells per high-power field was counted in 5–10 fields for each coded slide.

### Statistical analyses

Data are shown as the mean ± standard deviation (SD). Differences between two groups were assessed using Student’s t-test. Differences among three or more groups were evaluated by analysis of variance followed by Bonferroni multiple comparison tests.

## Results

### Iohexol-induced differential expression of Nox homologues in HK-2 cells

**Fig 2:**
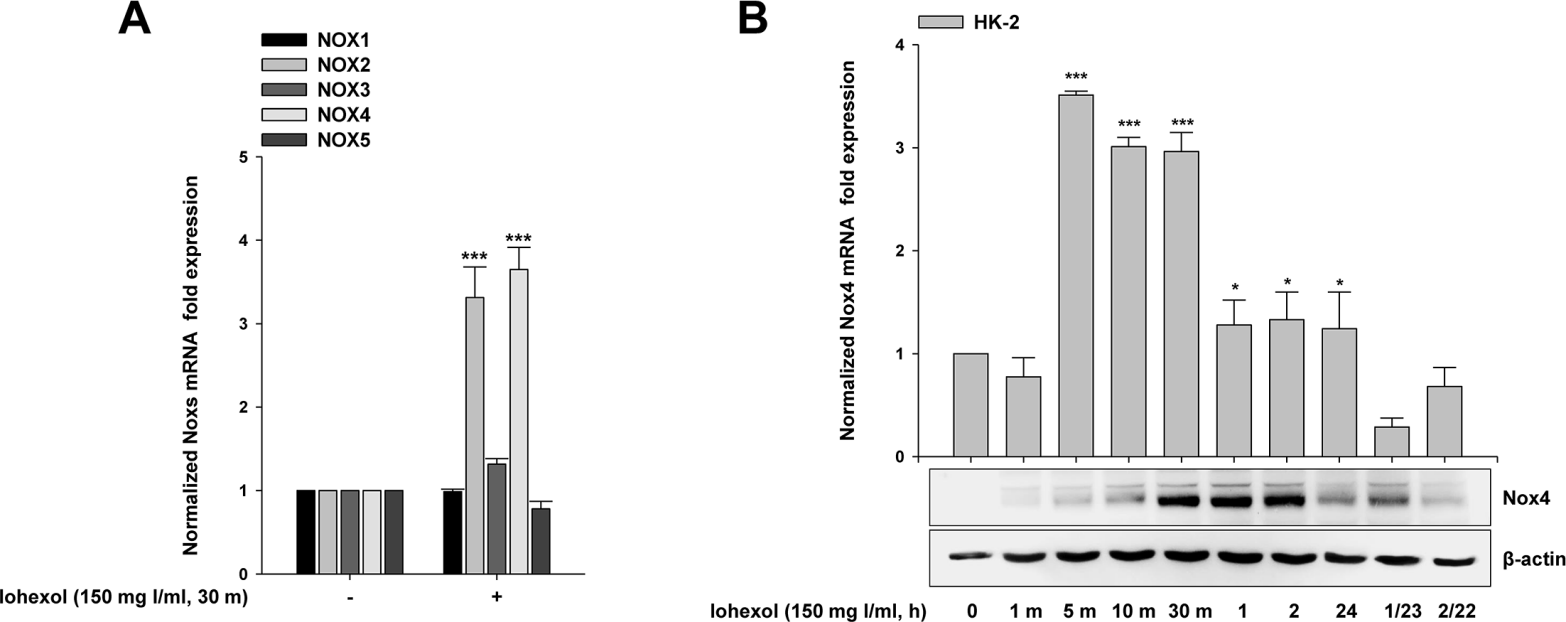
Iohexol-induced differential expression of Nox homologues and time-dependent Nox4 expression in HK-2 cells. **(A)** HK-2 cells were exposed to iohexol for 30 min. *Nox1*, *Nox2*, *Nox3*, *Nox4*, and *Nox5* mRNA levels after iohexol exposure were measured by quantitative real-time PCR. (**B)** HK-2 cells were exposed to iohexol for 0 min, 1 min, 5 min, 10 min, 30 min, 1 h, 2 h, and 24 h. For examination of the long-term viability of HK-2 cells after iohexol exposure, the medium containing iohexol was removed after 1 or 2 h and replaced with fresh serum-free medium for 23 and 22 h (1/23 and 2/22 h, respectively). Nox4 (*NOX4*) mRNA expression after iohexol exposure was measured by quantitative real-time PCR. Nox4 protein levels were measured by western blotting. Data are the mean ± SD (n = 5). *p < 0.05 and ***p < 0.001 versus the control.

We examined the effects of iohexol on Nox homologue (Nox1, Nox2, Nox3, Nox4, and Nox5) mRNA expression in cultured HK-2 cells. As shown in Fig 2, there were significant increases in Nox2 and Nox4 mRNA expression at 30 min after iohexol treatment (Fig 2A).

We further examined the effects of iohexol on cellular *Nox4* mRNA and protein expression in cultured HK-2 cells. As shown in Fig 2B, following iohexol treatment, there was a dramatic increase in *Nox4* expression within 5 min, as measured by real-time polymerase chain reaction (PCR). Iohexol also increased Nox4 protein levels, as measured by western blotting. After iohexol exposure, there was a gradual increase in Nox4 protein expression, which peaked at 2 h later and then decreased after 24 h, remaining higher than that in control (untreated) cells. For examination of the long-term viability of HK-2 cells after iohexol exposure, the medium containing iohexol was removed after 1–2 h and replaced with fresh serum-free medium. *Nox4* mRNA and protein levels at 23 and 22 h after removal of iohexol were also measured (1/23 h and 2/22 h). *Nox4* mRNA expression decreased to the level of the control, whereas the Nox4 protein level was still obviously increased at 23 and 22 h after removal of the iohexol stimulus (Fig 2B).

### Nox4 was involved in iohexol-induced apoptosis in human proximal tubular cells

**Fig 3:**
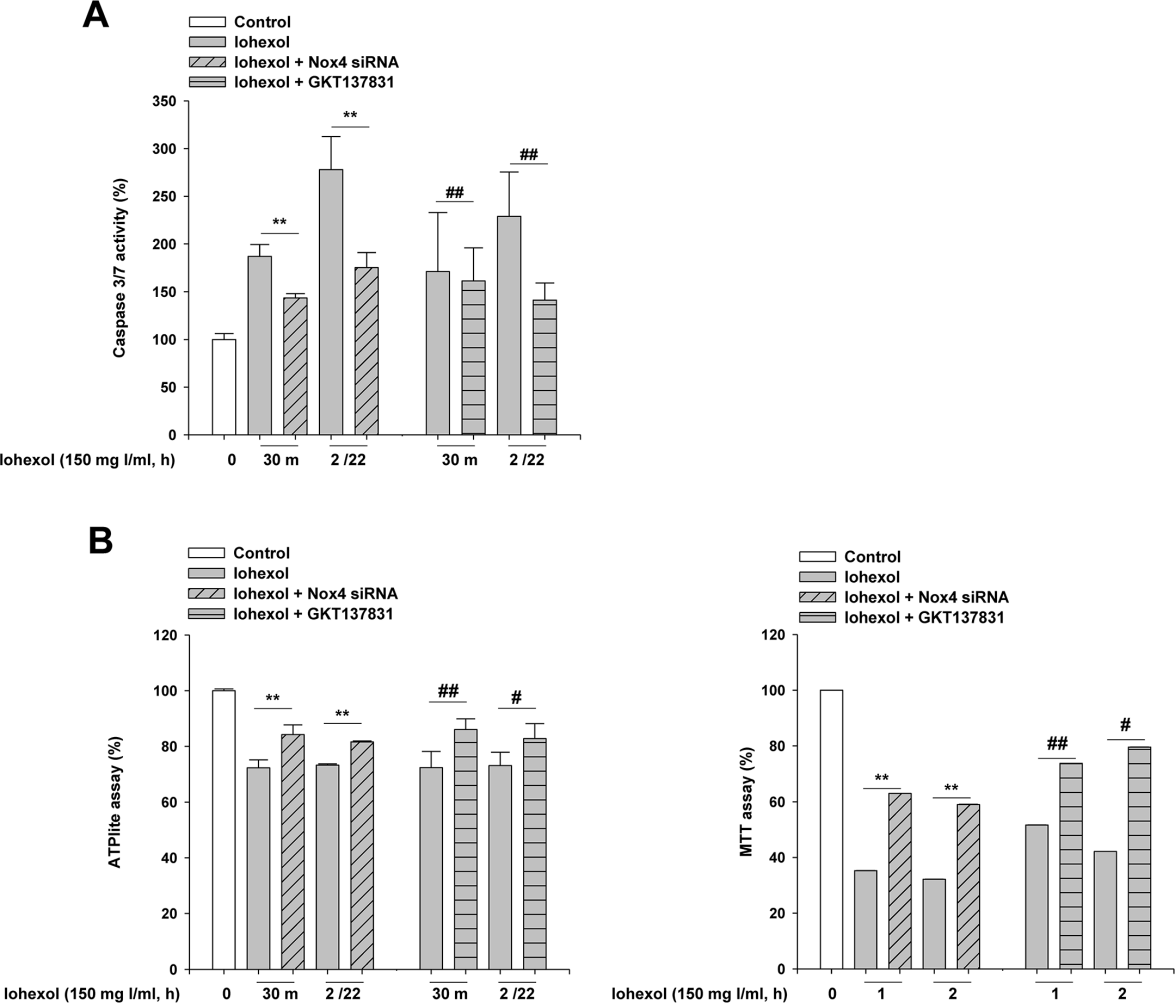
Effects of Nox4 knockdown and GKT137831 treatment on iohexol-induced apoptosis and cell death in HK-2 cells. HK-2 cells were cultured to 70–80% confluence, and iohexol was added. Cells were incubated with iohexol at the indicated concentrations for 30 min. For examination of the long-term viability of HK-2 cells after iohexol exposure, medium containing iohexol was removed after 2 h and replaced with fresh serum-free medium for 22 h (2/22 h). (**A**) The apoptotic response of HK-2 cells was assayed by measuring caspase 3/7 activity. (**B**) The viability of HK-2 cells was assayed using ATPlite and MTT assays. The data are the mean ± SD (n = 5). **p < 0.01 versus control and ^##^*p* < 0.01, ^###^*p* < 0.001 versus Iohexol treatment only.

To determine the role of Nox4 in iohexol-induced apoptosis, cells were transfected with *Nox4* small interfering RNA (siRNA) or control siRNA. After incubation of HK-2 cells with iohexol for 2 h, the medium containing iohexol was replaced with fresh serum-free medium, and caspase 3/7 activity was assessed. Silencing of Nox4 by transfection with *Nox4* siRNA led to a significant decrease in caspase 3/7 activity at 30 min after iohexol exposure and 22 h after iohexol removal (2/22 h). Pretreatment of HK-2 cells with GKT137831 at 20 μg/ml also abolished the apoptotic response induced by iohexol, as indicated by caspase 3/7 activation (Fig 3A). Moreover, silencing of Nox4 significantly improved the survival of HK-2 cells in response to iohexol, as measured by ATP assays and MTT assays. Pretreatment with GKT137831 replicated these renoprotective effects of *Nox4* knockdown (Fig 3B). *Nox4* knockdown efficiency is shown in S3A–S3C Fig for caspase 3/7 activity, ATPlite assay and MTT assay, respectively.

### Superoxide and ROS levels in human proximal tubular cells

**Fig 4:**
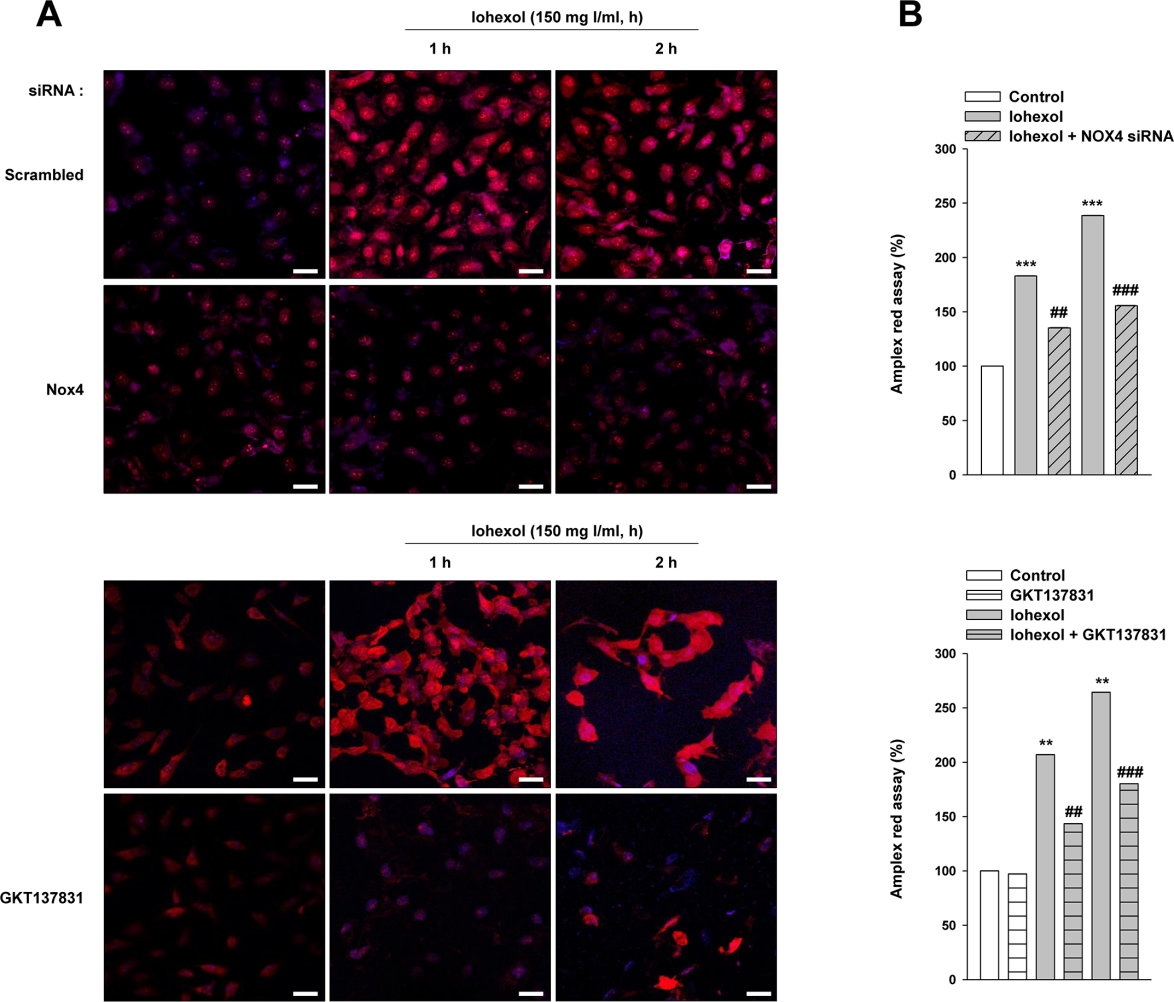
Effects of Nox4 on iohexol-induced ROS generation. Cells were exposed to iohexol for 0, 1, or 2 h with and without *Nox4* knockdown and GKT137831. (**A**) Confocal microscopic images of cells subjected to DHE staining. Original magnification, ×200; scale bar, 20 μm. (**B**) H_2_O_2_, a product of Nox4, was measured by Amplex red assay. The data are the mean ± SD. **p < 0.01, ***p < 0.001versus the control and ^##^*p* < 0.01, ^###^*p* < 0.001 versus iohexol treatment only.

We measured superoxide generation in HK-2 cells after iohexol exposure with or without *Nox4* knockdown and GKT137831 pretreatment using dihydroethidium (DHE) staining (Fig 4A). We also measured hydrogen peroxide generation using the Amplex red assay with or without *Nox4* knockdown and GKT137831 (Fig 4B). The presence of iohexol caused a significant increase in ROS production at 1 h and 2 h after iohexol exposure. Notably, the extent of iohexol induction of ROS levels was significantly decreased in the presence of *Nox4* silencing and GKT137831 pretreatment. The levels of glutathione peroxidase (GPx) and superoxide dismutase (SOD) were also measured with Immunoblotting after *Nox4* knockdown (S3D Fig). The levels of GPx and SOD were decreased after *Nox4* knockdown.

### Mitogen-activated protein kinases (MAPKs) mediated redox-sensitive, iohexol-induced apoptosis in proximal tubular cells

**Fig 5:**
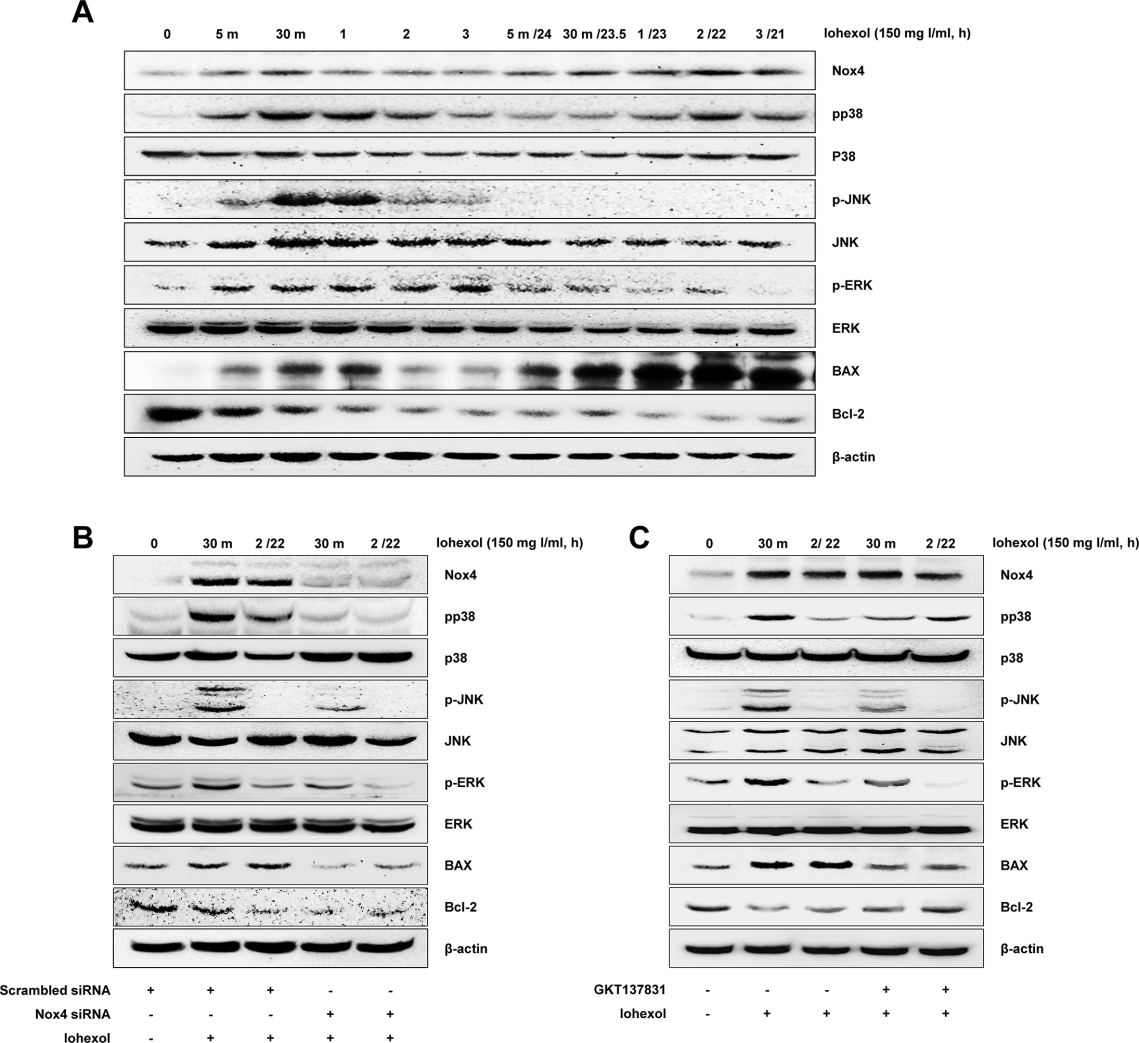
Western blot analysis showing intracellular Nox4 signaling in iohexol-induced apoptosis in HK-2 cells. HK-2 cells were incubated with iohexol for the indicated times (0 min, 5 min, 30 min, 1 h, 2 h, or 3 h). For examination of the long-term viability of HK-2 cells after iohexol exposure, the medium containing iohexol was removed after 5 min, 0.5 h, 1 h, 2 h, or 3 h and then replaced with fresh serum-free medium for 24 h, 23.5 h, 23 h, 22 h, or 21 h (5 min/24 h, 0.5/23.5 h, 1/23 h, 2/22 h or 3/21 h). (**A**) Effects of Nox4 on phosphorylation of MAPK family members (p38, JNK, and ERK), bax, bcl-2, and p65. (**B** and **C**) Effects of Nox4 knockdown and GKT137831 pretreatment on iohexol-induced apoptosis.

To elucidate the signaling mechanisms of iohexol- and Nox4-mediated apoptosis, we analyzed the activation of redox-sensitive MAPK pathways (p38, c-Jun N-terminal kinase [JNK], and extracellular signal-regulated kinase [ERK] pathways). Incubation of HK-2 cells with iohexol at a concentration of 150 mg I/ml caused increasing phosphorylation of p38, JNK, and ERK within 5 min after iohexol exposure (Fig 5A and S4 Fig).

For examination of the long-term viability of HK-2 cells after iohexol exposure, after HK-2 cells were incubated with iohexol for 5 min, 30 min, 1 h, 2 h, and 3 h, iohexol-containing medium was replaced with fresh serum-free medium, and the phosphorylation and activation of MAPKs and apoptosis signaling pathways were assessed. Among MAPKs, only the levels of phospho-p38 remained high after removal of iohexol. The level of Bax also remained elevated after iohexol removal (Fig 5A).

To determine whether the activation of MAPK pathways was redox- and Nox4-dependent, we knocked down Nox4 using Nox4 siRNA and found that Nox4 knockdown reduced iohexol-induced phosphorylation of p38, JNK, and ERK at 30 min after iohexol exposure. The level of Bax was also significantly decreased at 30 minutes after addition of iohexol in Nox4-knockdown HK-2 cells compared to that in iohexol-exposed control cells. For examination of the long-term viability of HK-2 cells after iohexol exposure, after incubation of HK-2 cells with iohexol for 2 h, the iohexol-containing medium was replaced with fresh serum-free medium, and the phosphorylation and activation of MAPKs and apoptosis signaling pathways were assessed. Silencing of Nox4 reduced the phosphorylation of p38 and the expression of Bax at 22 h after iohexol removal compared to that in control cells under the same conditions (Fig 5B). Moreover, pretreatment with GKT137831 also replicated these results (Fig 5C).

**Fig 6:**
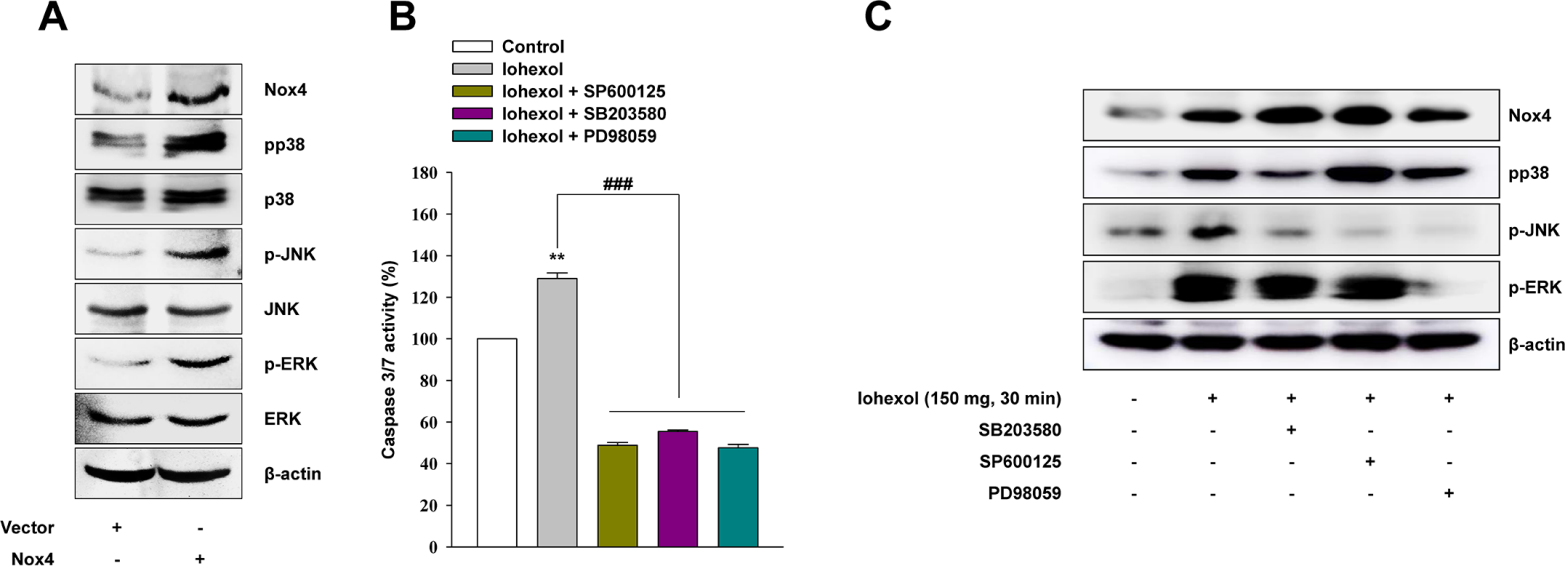
Effects of Nox4 overexpression mediated by HA vector-based gene transfer on HK-2 cell apoptosis. Virus expressing the HA vector was used as a control. (**A**) Western blot analysis of the effects of HA vector-Nox4 on p38, JNK, and ERK phosphorylation. (**B**) Effects of inhibitors of p38 (SB203580), JNK (SP600125), and ERK (PD98059) on cell apoptosis, as measured by caspase 3/7 activity assays. (**C**) Levels of Nox4 protein were measured after inhibition of p38 (SB203580), JNK (SP600125), and ERK (PD98059). The data are the mean ± SD (n = 5). **p < 0.01 versus the control and ^###^*p* < 0.001 versus iohexol treatment only.

We further confirmed the relationship between Nox4 and MAPKs by overexpression of Nox4 in HK-2 cells. Nox4 overexpression by transfection with Nox4 cDNA increased p38, JNK, and ERK phosphorylation (Fig 6A). Notably, inhibition of p38, JNK, and ERK with SB203580, SP600125, and PD98059, respectively, decreased caspase3/7 activity (Fig 6B). However, inhibition of p38, JNK, and ERK with SB203580, SP600125, and PD98059, respectively, showed no significant reduction of Nox4 after iohexol exposure (Fig 6C).

### Effect of GKT137831 on attenuation of renal dysfunction and oxidative stress in CIAKI in mice

We evaluated the effects of iohexol and the Nox4 inhibitor GKT137831 *in vivo*. Compared with the control group, the levels of BUN were significantly higher in the iohexol and GKT137831 pretreatment groups (*p < 0.05). However, plasma creatinine levels were not different between the groups. For oxidative stress measurement, serum levels of SOD were evaluated. Serum SOD levels were significantly increased in the GKT137831 pretreatment group when compared with that in the iohexol group (Table 1).

**Table 1:** Renal functional and renal oxidative stress parameters in the study groups (mean ± SD).

	Groups		
Parameter	G1 (n = 8) Control	G2 (n = 8) Iohexol	G3 (n = 8) Iohexol + GKT137831 (40 mg/kg)
**BUN (mg/dL)**	22.0 ± 1.4	45.4 ± 24.8*	39.0* ± 9.4
**Cr (mg/dL)**	0.16 ± 0.06	0.21 ± 0.037	0.22 ± 0.05
**SOD activity (μg/ml)**	90.2±1.7	89.4±1.3	96.3 ± 2.3^#^

The data are the mean ± SD (n = 8).

*p < 0.05

**p < 0.01

***p<0.001 versus the control and

^#^*p* < 0.05

^##^*p* < 0.01

^###^*p* < 0.001 versus the iohexol.

Abbreviations: G1, group 1; G2, group2; G3, group 3; BUN, blood urea nitrogen; Cr, creatinine; NGAL, neutrophil gelatinase-associated lipocalin; SOD, superoxide dismutase.

### Effect of GKT137831 on renal histological alterations caused by iohexol

**Fig 7:**
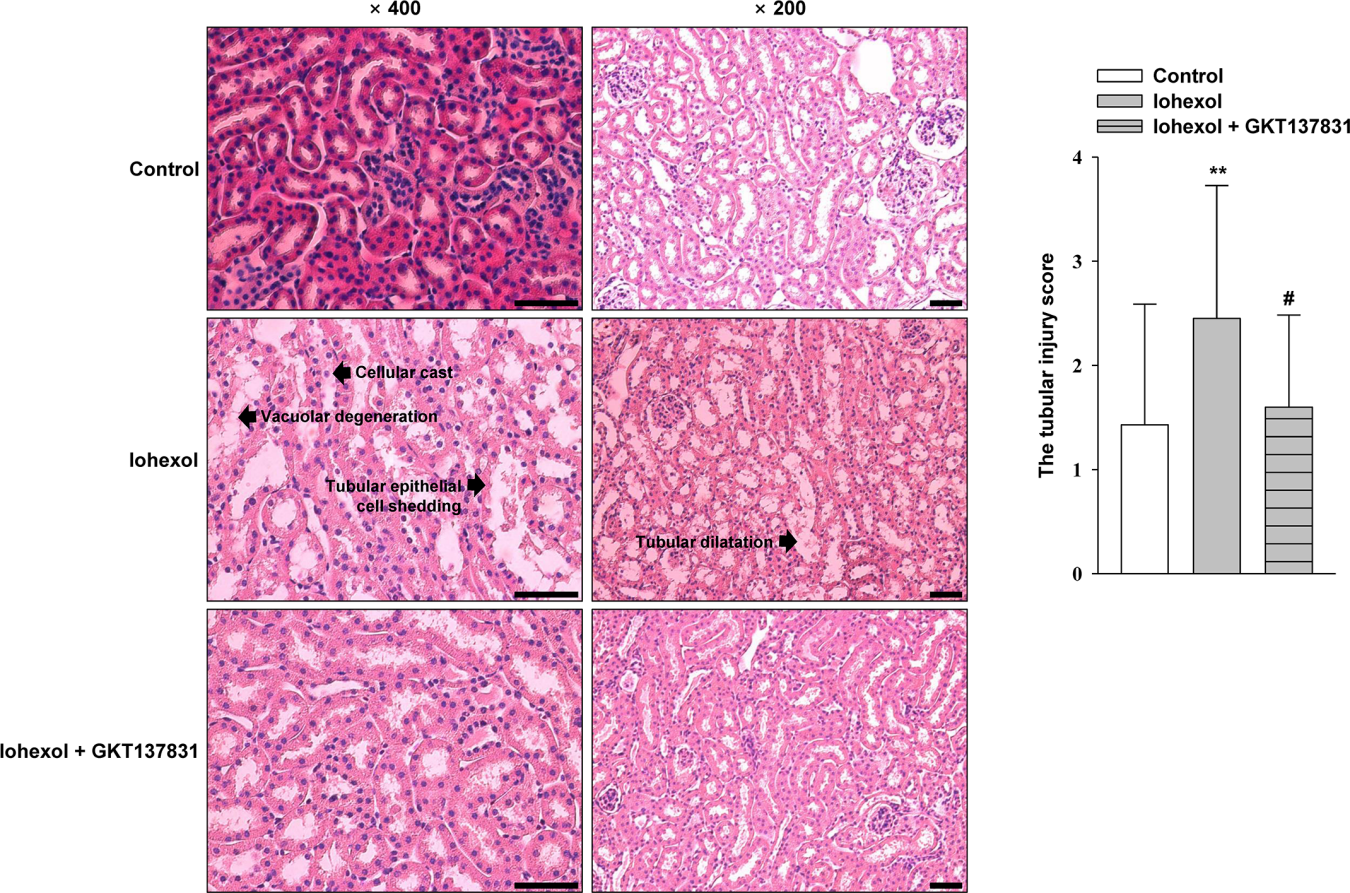
Representative photomicrographs of hematoxylin & eosin (H&E)-stained kidney sections and a semiquantitative scoring analysis of tubular injury at 24 h after intraperitoneal injection of iohexol with or without GKT137831 pretreatment. The marked tubular injury caused by iohexol was diminished as the result of GKT137831 treatment. For semiquantitative analysis of morphological changes, 10 high-magnification (×200) fields for the cortex and the outer stipe of the outer medulla in ten mice of each group were randomly selected. Magnifications, ×400 and ×200; scale bar, 100 μm.

Our histopathologic examination of iohexol-injected mice showed evidence of tubular epithelial cell shedding, basement membrane nudity, vacuolar degeneration of tubular epithelial cells, protein casts, tubular dilation, loss of tubular brush borders, and necrosis of partial tubular epithelial cells. However, in GKT137831-pretreated mice, only tubular epithelial cell degeneration was observed. Light microscopic examination and semiquantitative analysis demonstrated that the tubular pathological scores in the iohexol group were significantly higher than those in control animals (^**^p < 0.01). Pretreatment with GKT137831 significantly attenuated the development of these lesions, as indicated by tubular pathological scores (*p*^*#*^ < 0.05) (Fig 7 and S5 Fig).

### Nox homologue expression after iohexol exposure

Immunohistochemistry (IHC) was performed for expression of Nox 4 and other Noxs (Nox1 and Nox2) *in vivo* (S6 Fig). The expression of Nox4 and Nox1 was not significantly different among control, iohexol and GKT pretreatment groups. Expression of Nox2 was significantly increased in the iohexol group and significantly decreased in the GKT pretreatment group. Nox4 was expressed in the nucleus and cytoplasm, whereas Nox2 was expressed in the cellular membrane. Both were present mostly in proximal tubular epithelial cells.

### Effect of GKT137831 on renal ROS induced by iohexol

**Fig 8:**
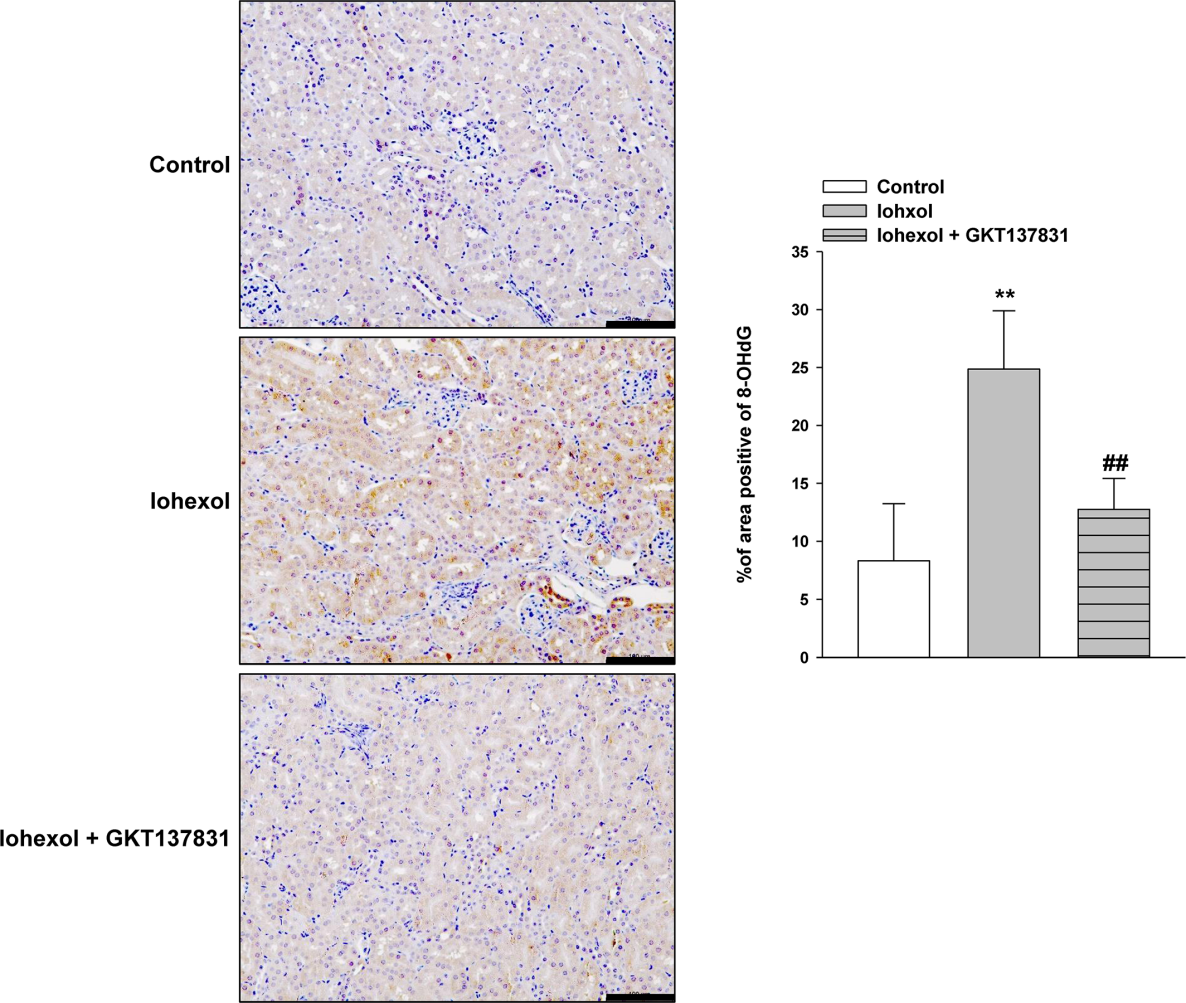
Amelioration of oxidative stress in rats with iohexol-induced nephrotoxicity treated with GKT137831. Representative photomicrographs of immunostaining of renal 8-OHdG (8-hydroxy-2’-deoxygenase) at 24 h after intraperitoneal injection of iohexol in control mice or mice treated with iohexol with or without GKT137831 pretreatment. Image analysis of the extent and intensity of staining was performed. Data are presented as the mean ± SD (n = 4–5). **p < 0.01 versus the control and ^##^*p* < 0.01 versus iohexol treatment only. Magnification, ×200; scale bar, 100 μm.

We investigated whether GKT137831 decreased the ROS levels induced by iohexol. Kidney sections at 24 h after iohexol administration were immunostained to detect the presence of an oxidized derivative of deoxyguanosine (8-OHdG). In the iohexol group, the number of superoxide 8-OHdG-positive renal tubules was markedly increased compared to that in the control group. In contrast, GKT137831 administration clearly decreased the number of superoxide 8-OHdG-positive cells compared with that in the control group (Fig 8).

### Effect of GKT137831 on mitogen-activated protein kinases (MAPKs), pro-apoptotic and pro-survival protein changes caused by iohexol

Whole-tissue lysates were prepared from mouse kidneys excised from animals exposed to iohexol and subjected to western blotting, and the results are shown in S7A and S7B Fig for MAPKs and apoptotic signals, respectively. Phospho-p38/p38, phospho-pJNK/pJNK and phospho-ERK/ERK levels were significantly decreased in the GKT137831 pretreatment group (S7A Fig). Iohexol caused a dramatic increase in the level of the pro-apoptotic protein Bax. However, the level of the pro-survival protein Bcl-2 also decreased after iohexol exposure. The levels of Bax were significantly suppressed in the presence of GKT137831, and GKT137831 also significantly increased the levels of Bcl-2 (S7B Fig).

### Effect of GKT137831 on renal tubular apoptosis and necrosis caused by iohexol

**Fig 9:**
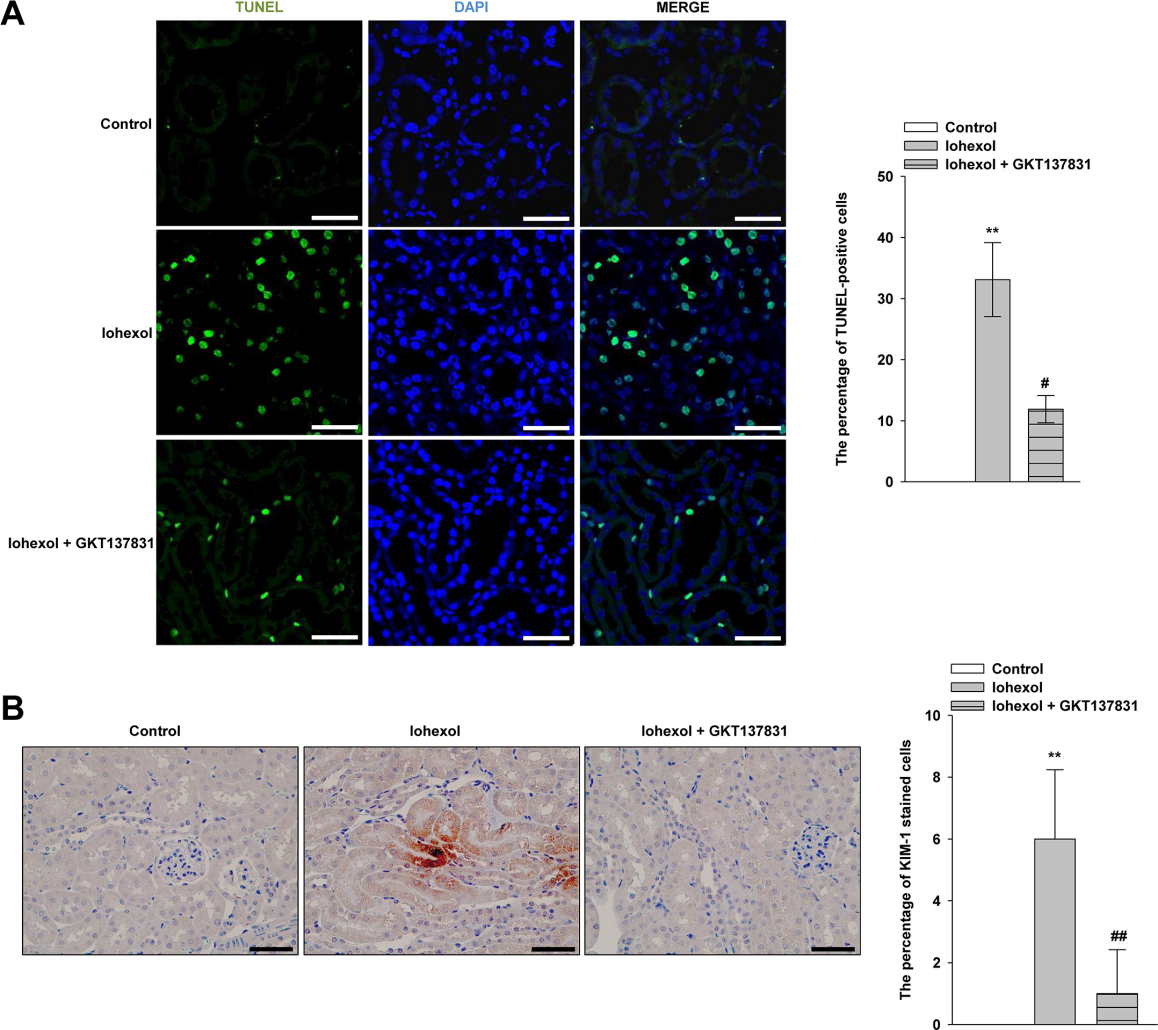
**Effect of GKT137831 on renal tubular apoptosis and necrosis caused by iohexol** TUNEL staining **(A)** and immunostaining of KIM-1 **(B)** in control mice or mice with iohexol-induced nephropathy, treated with or without GKT137831. Representative confocal micrographs of TUNEL- **(A)** and KIM-1- **(B)** stained kidney sections at 24 h after intraperitoneal injection of 4000 mg/kg iohexol in control mice, mice treated with iohexol, and mice treated with iohexol after GKT137831 pretreatment. Data are presented as the mean ± SD (n = 4–5). **p < 0.01 versus the control and ^##^*p* < 0.01 versus iohexol treatment only. Image analysis of the extent and intensity of staining was performed. Magnification, ×200; scale bar, 100 μm.

TUNEL immunostaining was performed to evaluate renal tubular apoptosis (Fig 9A). Kidneys from the iohexol group showed a marked increase in the number of TUNEL-positive tubular epithelial cells. In contrast, the number of TUNEL-positive cells in the GKT137831-treated group was markedly decreased compared with that in the iohexol group. We also performed immunostaining for kidney injury molecule-1 (KIM-1) to detect acute tubular necrosis to determine the effect of GKT137831 in CIAKI mice. The number of KIM-1-positive cells was significantly increased after iohexol injection, and this was reversed by GKT137831 treatment (Fig 9B).

## Discussion

In this study, we showed that exposure of renal proximal tubular cells to CM resulted in significant increases in Nox4 expression and activity with increased ROS. Knockdown of *Nox4* transcription and pretreatment with the Nox1/4 inhibitor GKT137831 resulted in decreased intracellular oxidative stress and ROS-mediated apoptosis in renal proximal tubular cells. In addition, pretreatment with GKT137831 attenuated proximal tubular cell injury in an animal model of CIAKI.

Previous studies have suggested that Nox4 expression in the kidney is associated with the development and progression of chronic kidney disease, particularly diabetic nephropathy with renal fibrosis [11–14]. However, its roles in AKIs have not been extensively investigated. In this study, we showed that Nox4 expression was markedly increased after iohexol exposure using real-time PCR and western blotting. Recently Netti *et al*. reported that Nox4 protein expression was increased after CM exposure [19]. However, the pathological role of Nox4 in CIAKI was not examined thoroughly. We demonstrate for the first time the role of Nox4 as a key mediator of cellular apoptosis after CM exposure via the phospho-p38 MAPK pathway.

Although the pathogenic mechanisms of CIAKI are controversial, previous studies suggested that oxidative stress and tubular apoptosis play an important role in the development of CIAKI [4, 19–23]. Similar to previous studies, in this study, exposure of proximal tubular cells to CM induced oxidative stress, as indicated by DHE staining and amplex red assay. In vivo, the accumulation of 8-OHdG increased after CM exposure.

Several regimens, such as N-acetylcysteine (NAC; a reactive oxygen scavenger), ascorbic acid, tocopherol, and atorvastatin, have been evaluated for reducing reactive oxygen species (ROS) levels to prevent CIAKI [24–27]. However, the preventive effects have been equivocal in many clinical studies [28–31]. Therefore, we hypothesized that direct reduction of ROS production may be more effective than inhibition of downstream signaling pathways to prevent ROS-associated CIAKI. In a previous study, protective effects of apocynin were demonstrated in a CIAKI rat model [32]. Apocynin has been shown to prevent the translocation of p47phox to Nox2 in leukocytes, monocytes, and endothelial cells [33, 34]. However, the activity of Nox4 is determined by mRNA expression; thus, the efficacy of apocynin in reducing ROS levels may not be sufficient in the kidney. In this study, the direct inhibition of ROS production by inhibiting Nox4 markedly attenuated CM-induced ROS and caspase 3/7 activity in renal proximal tubular cells. Furthermore, these changes resulted in a reduction of the number of apoptotic cells in an animal model of CIAKI.

Angiotensin II, insulin, tumor necrosis factor (TNF) α, endoplasmic reticulum stress, shear stress, carotid artery balloon injury, hypoxia, and ischemia are known as inducers of Nox4 in various cell types [35]. CM also has direct vasoconstrictor effects and exacerbates vascular ischemia because the vasoconstrictor hormones (e.g., rennin, endotholin, and adenosins) increase and the vasodilator hormones (e.g., prostaglandin and nitric oxide) decrease [36]. These mediators could be involved in Nox4 expression after iohexol exposure.

Recently, Nlandu-Khodo et al. [37] reported that Nox4 is anti-apoptotic in ischemia/reperfusion injury. These findings contradict the observations identifying Nox4 as a mediator of kidney disease. Despite the beneficial signaling role of H_2_O_2_ in various cells, it is still true that excessive concentrations of H_2_O_2_ induce inflammation, fibrosis, apoptosis, and even necrosis. Thus, whether Nox4 is harmful or beneficial is primarily dependent on the amount present [38]. Different levels of oxidative stress could lead to different effects on kidney injury in hypoxia.

ROS trigger apoptosis via multiple mechanisms; among these mechanisms, MAPKs are well-documented redox-sensitive mediators involved in cell apoptosis [39–42]. In this study, we found that phosphorylation of p38, JNK, and ERK was highly increased at approximately 1 h after iohexol exposure. Although the phosphorylation levels of JNK and ERK gradually decreased with time after removal of CM from HK-2 cells, the elevated levels of phospho-p38 were maintained after CM removal. Therefore, we speculate that p38 may play a more central role in long-term cell death/survival in HK-2 cells treated with CM. This observation is consistent with other studies showing similar pro-apoptotic roles of p38 in CIAKI [43, 44]. However, the mechanisms through which ROS activate p38 in proximal tubular cells are not clear. In response to increased ROS production, activation of p38 has been shown to directly affect the phosphorylation and activation of some pro-apoptotic Bcl-2 family proteins, such as Bim and Bax [39]. Consistent with this, we found that the increase in Bax levels after CM exposure was significantly diminished after Nox4 inhibition. Inhibition of ERK after Nox4 overexpression with cDNA transfection rescued the cells from apoptosis in this study. Persistent activation of the p38 and JNK signaling pathways has been shown to mediate cellular apoptosis, whereas the ERK signaling pathway plays a key role in cellular proliferation, migration, and invasion [45]. In contrast, sustained activation of ERK induces expression of gene products related to death-promoting activity [46]. Nox4 overexpression in HK-2 cells by transfection with Nox4 cDNA in this study could have resulted in the sustained activation of ERK and increased apoptosis. However, after CM exposure, the phosphorylation of ERK was transiently increased at 1 h and then decreased gradually, similar to the phosphorylation of JNK. Accordingly, the intracellular signaling role of phospho-ERK after CM exposure could be different from that induced by Nox4 overexpression by transfection with Nox4 cDNA.

Another interesting point in this study was the interaction between Nox2 and Nox4 in CIAKI *in vitro* and *in vivo*. *Nox2* and *Nox4* mRNA levels were significantly increased at 30 min after iohexol exposure *in vitro*. Although Nox4 expression was decreased at 24 h after iohexol exposure *in vitro* and *in vivo*, Nox2 protein levels remained high for 24 h *in vivo*. The levels of Nox2 protein were significantly decreased after inhibition of Nox4 with GKT137831. These changes occurred concurrently with improvement of CIAKI. Recently, it was reported that Nox4-derived H_2_O_2_ activates Nox2 to promote mitochondrial ROS production via pSer36-p66Shc in endothelial cells [47]. The Nox4/Nox2 axis could be involved in the amplification of ROS production and CKAKI progression.

In GKT137831-treated animals, tubular pathological changes and apoptosis associated with iohexol were reduced with decreasing oxidative stress. However, the improvement of biochemical markers (BUN, creatinine and MDA) after pretreatment of GKT137831 was unclear. The levels of creatinine and MDA were not different between the groups, and the levels of BUN were lower in the GKT137831 pretreatment group than in the iohexol group, but the improvement was not significant. In terms of possible reasons for these effects, first, the sample size of 8 mice might not be sufficient to detect a difference in biochemical markers. Second, compared to in vitro, in vivo experiments have high variation among individuals due to cell-to-cell interactions and differences of renal blood flow and concentrations of CM in the renal tubule. Third, various mechanisms usually contribute to CIAKI, not only Nox4. Fourth, 40 mg/kg GKT137831 was administered for 5 days before iohexol injection. However, few reports have investigated the prevention of AKI by GTK137831; therefore, it is not clear whether the dosage and duration used in this study were adequate for reno-protection in CIAKI. Thus, further studies are needed to solve these problems. Despite these limitations, to the best of our knowledge, this is the first study investigating the oxidative role of Nox4 and the protective effects of Nox4 inhibition in CIAKI.

**Fig 10:**
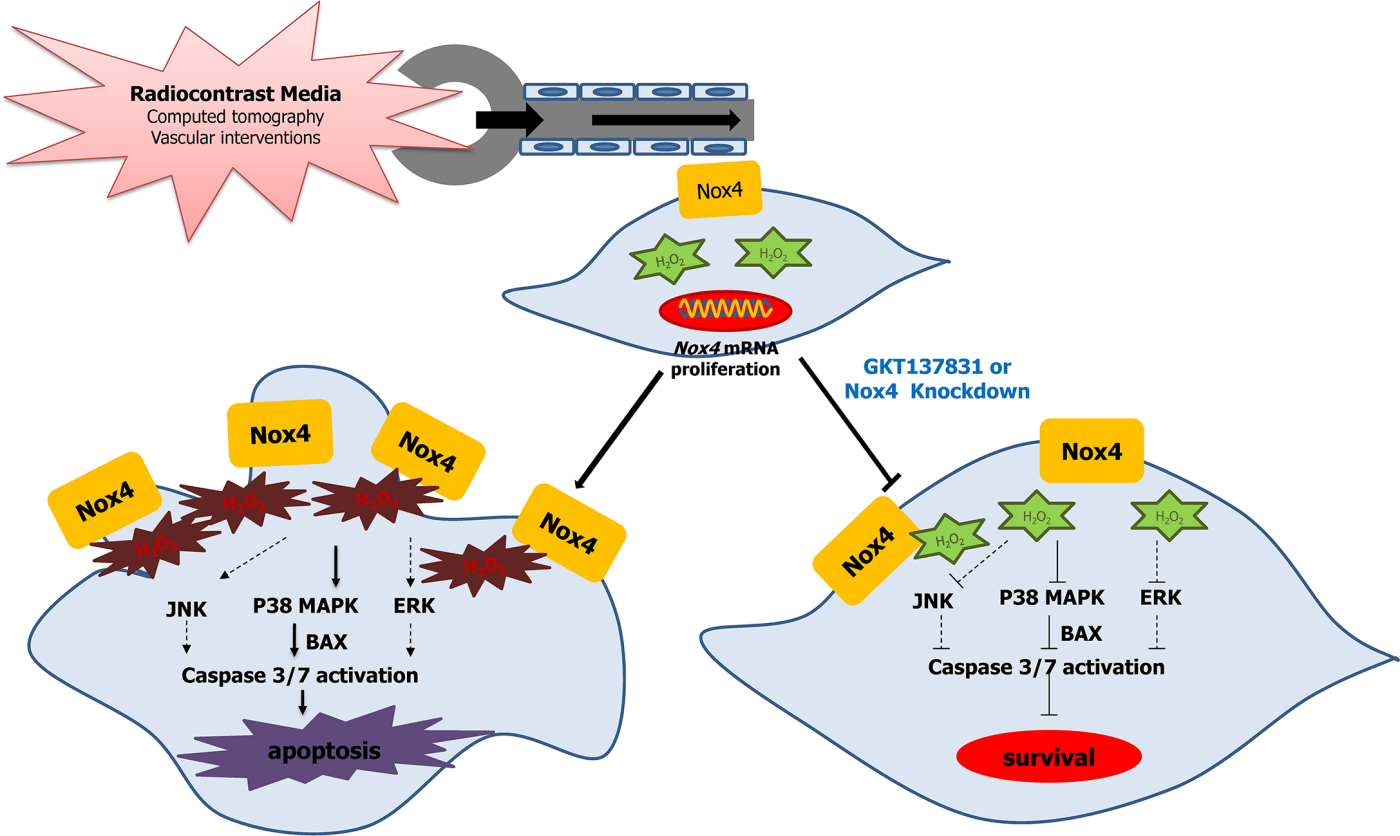
Proposed model of radiocontrast medium-induced acute kidney injury enhanced by oxidative stress caused by activation of Nox4 in HK-2 cells. Radiocontrast medium induces proximal tubular cell apoptosis through increased oxidative stress by Nox4 activation. Inhibition of Nox4 by genetic knockdown of Nox4 or by treatment with GKT137831 (specific Nox1/4 inhibitor) attenuated radiocontrast-induced proximal tubular cell apoptosis through reduction of MAPK (especially p38 MAPK subfamily) phosphorylation and Bax levels.

In conclusion, based on the findings obtained from our current experiments, we propose that CM upregulates Nox4 and causes increased ROS production. The enhanced ROS production triggers induction of MAPKs, especially p38. The silencing of Nox4 expression and pharmacological inhibition of Nox4 activity further support a model whereby CIAKI is dependent upon Nox4 activation (Fig 10). These results suggest that direct targeting of Nox4 may be helpful in the prevention of ROS-mediated CIAKI and could provide novel potential strategies for reducing AKI-related mortality.

## Supporting information

S1 Fig Chemical structure of the dual Nox1/4 inhibitor GKT137831. (TIFF)

S2 Fig Dose-dependent effects of Nox4 siRNA. Cells were used to measure mRNA levels of Nox4 by real-time PCR. Nox4 silencing efficiency was identified by dose dependency experiments. Data are presented as the mean ± SD (n = 4–5). **p < 0.01 and ***p < 0.001 versus the control.
(TIFF)

S3 Fig Nox4 knockdown efficiency. Cells were used to measure mRNA levels of Nox4 by real-time PCR. Nox4 silencing efficacy was confirmed for study of caspase 3/7 activity, ATPlite assay, and ROS detection (**A**, **B** and **C**). The levels of GPx and SOD protein were measured with western blotting after Nox4 knockdown (**D**). Data are presented as the mean ± SD (n = 4–5). ***p < 0.001 versus the control.
(TIFF)

S4 Fig Western blot analysis showing the intracellular signaling of Nox4 in iohexol-induced apoptosis in HK-2 cells. HK-2 cells were incubated with iohexol for the indicated times (0, 1, 2, or 24 h). (**A** and **B**) Effects of Nox4 knockdown and GKT137831 pretreatment on iohexol-induced apoptosis.
(TIFF)

S5 Fig Tubular injury caused by iohexol was diminished by pretreatment with GKT137831. Representative photomicrographs of Periodic acid-Schiff stain- (PAS) stained kidney sections are presented for the control group, iohexol group, and iohexol + GKT137831 group. Figures are representative of eight mice in each group. Magnifications: x 400 in A through C; x 200 in D through F; Magnifications, ×400 and ×200; scale bar, 100 μm.
(TIFF)

S6 Fig Nox homologue expression in the CIAKI mouse model. We performed immunohistochemistry (IHC) to determine the expression of Nox4 and other Noxs (Nox1 and Nox2) expression in vivo. The expression of Nox4 and Nox1 was not significantly different among control, iohexol and GKT pretreatment rats. Expression of Nox2 was significantly increased in iohexol rats and significantly decreased in GKT pretreatment rats. The data are the mean ± SD (n = 5). *p < 0.05 versus the control and ^###^*p* < 0.001 versus iohexol treatment only.
(TIFF)

S7 Fig Western blot analysis showing the effect of GKT-137831 on the MAPKs, pro-apoptotic and pro-survival proteins in mice with iohexol-induced nephropathy. The protein levels of MAPKs (phospho-p38, p38, phospho-JNK, JNK, phosphor-ERK, ERK (**A**), Bax, and Bcl-2 (**B**) in kidney lysates from different groups were examined. Data are presented as the mean ± SD (n = 4–5). *p < 0.05 versus the control and ^#^*p* < 0.05 versus Iohexol treatment only.
(TIFF)
