# Supplementary figures and images for: Oxidative stress caused by activation of NADPH oxidase 4 promotes contrast-induced acute kidney injury
Source: PLoS One. 2018 Jan 12;13(1):e0191034. doi: 10.1371/journal.pone.0191034 (PMC5766150; doi:10.1371/journal.pone.0191034)

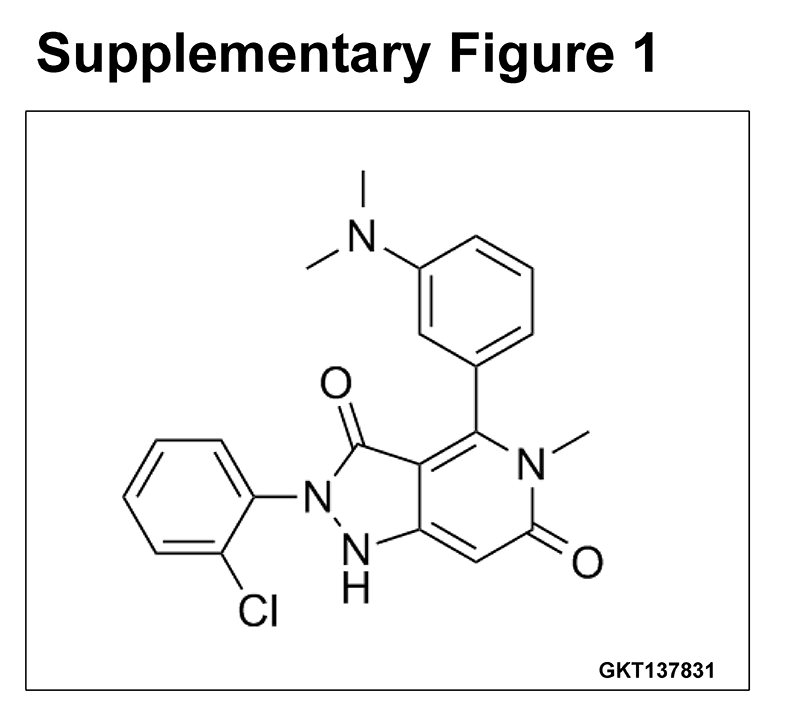

Supplement: S1 Fig — (TIFF) [file pone.0191034.s001.tiff]

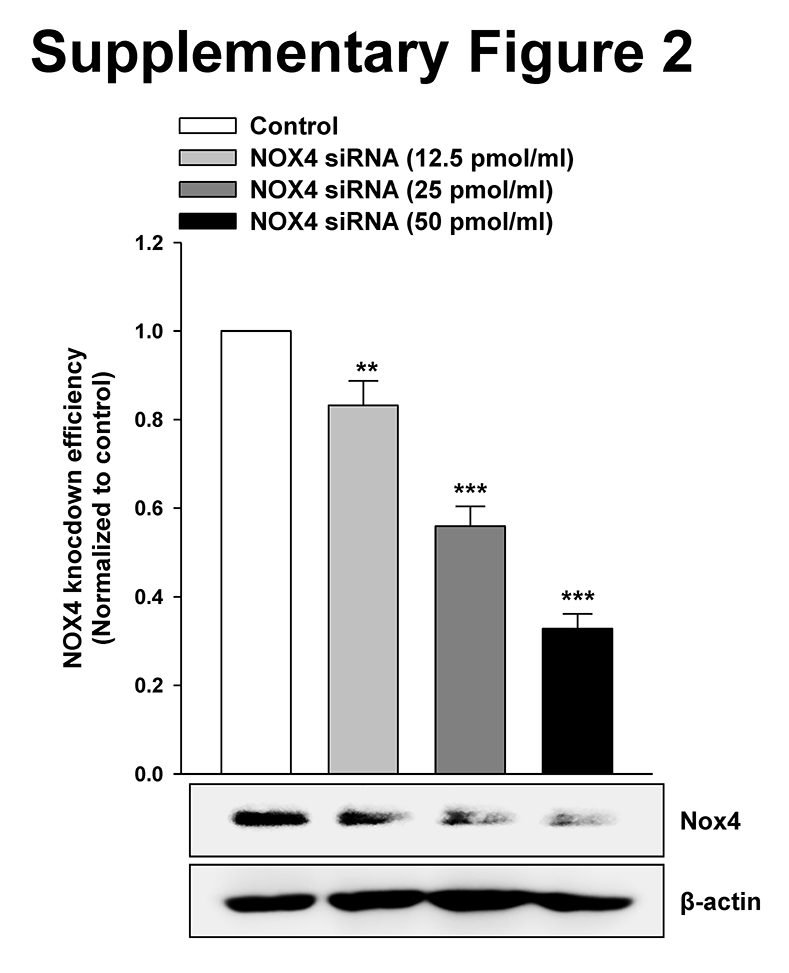

Supplement: S2 Fig — Cells were used to measure mRNA levels of Nox4 by real-time PCR. Nox4 silencing efficiency was identified by dose dependency experiments. Data are presented as the mean ± SD (n = 4–5). **p < 0.01 and ***p < 0.001 versus the control. (TIFF) [file pone.0191034.s002.tiff]

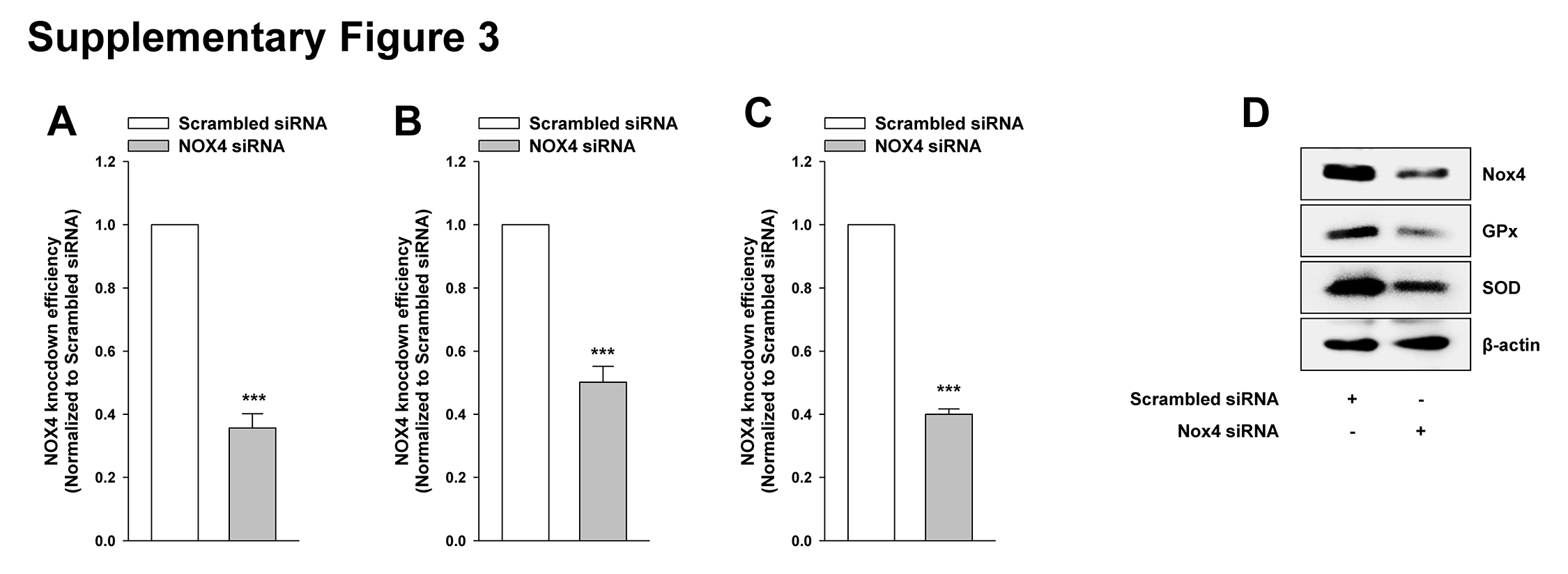

Supplement: S3 Fig — Cells were used to measure mRNA levels of Nox4 by real-time PCR. Nox4 silencing efficacy was confirmed for study of caspase 3/7 activity, ATPlite assay, and ROS detection (A, B and C). The levels of GPx and SOD protein were measured with western blotting after Nox4 knockdown (D). Data are presented as the mean ± SD (n = 4–5). ***p < 0.001 versus the control. (TIFF) [file pone.0191034.s003.tiff]

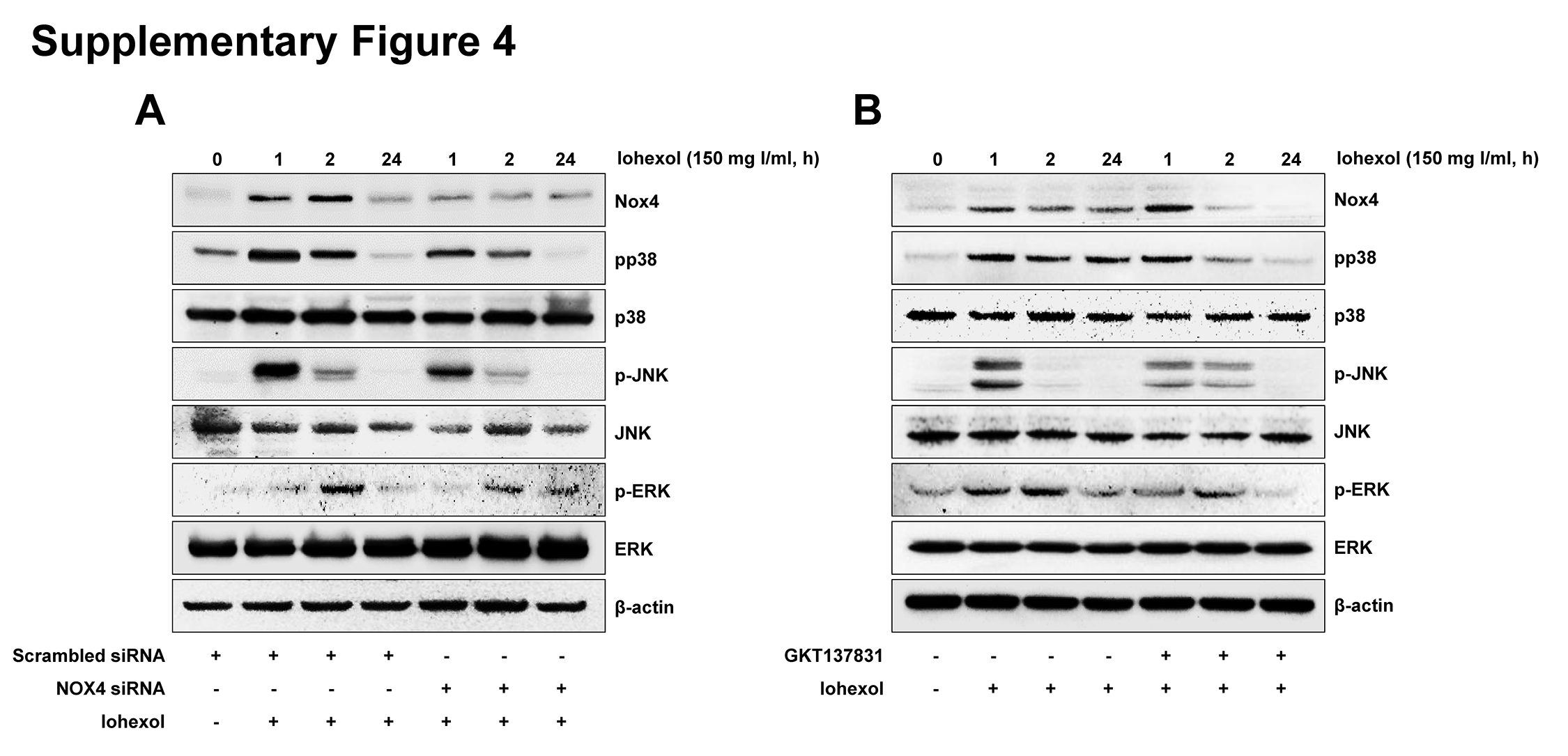

Supplement: S4 Fig — HK-2 cells were incubated with iohexol for the indicated times (0, 1, 2, or 24 h). (A and B) Effects of Nox4 knockdown and GKT137831 pretreatment on iohexol-induced apoptosis. (TIFF) [file pone.0191034.s004.tiff]

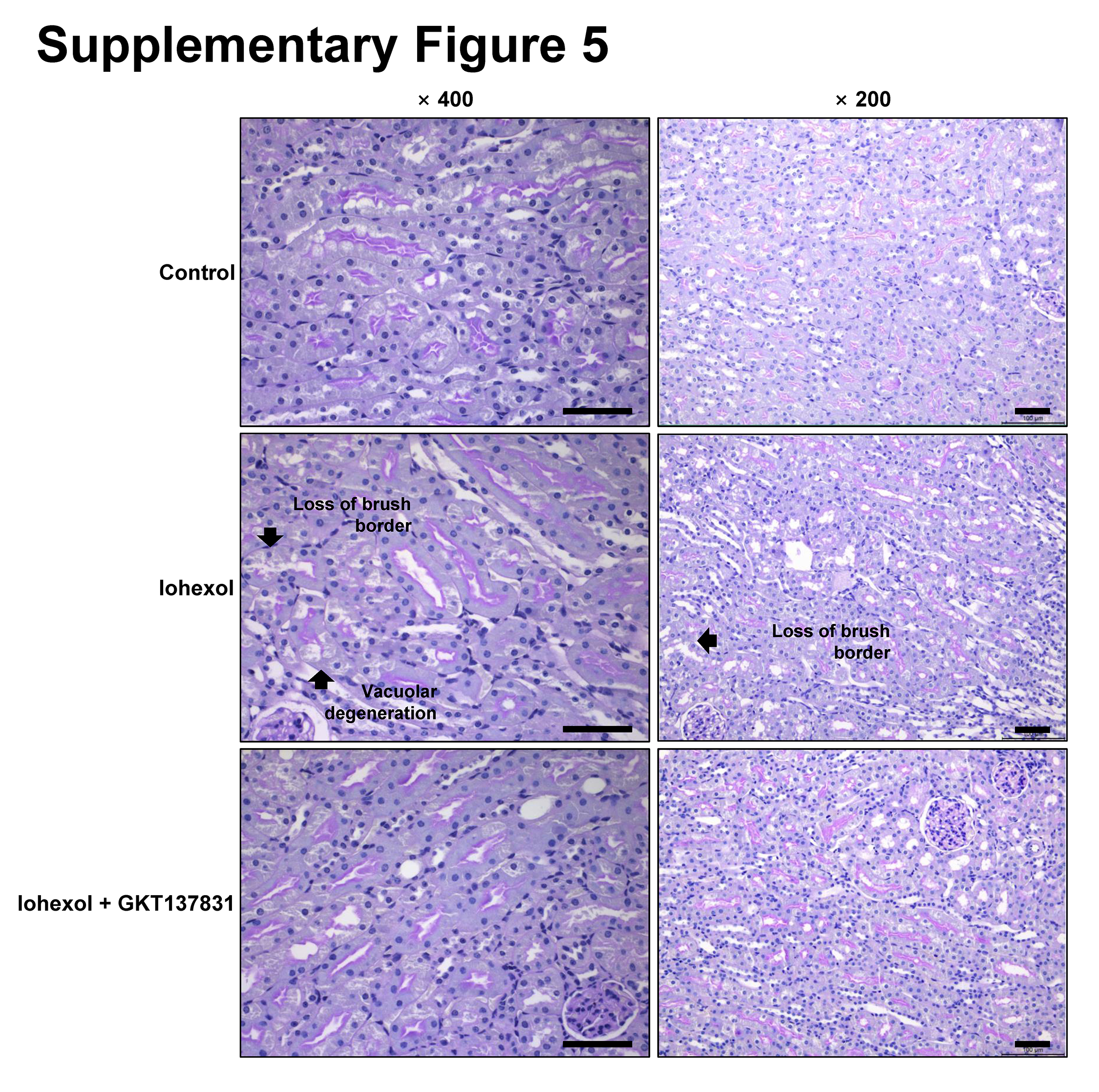

Supplement: S5 Fig — Representative photomicrographs of Periodic acid-Schiff stain- (PAS) stained kidney sections are presented for the control group, iohexol group, and iohexol + GKT137831 group. Figures are representative of eight mice in each group. Magnifications: x 400 in A through C; x 200 in D through F; Magnifications, ×400 and ×200; scale bar, 100 μm. (TIFF) [file pone.0191034.s005.tiff]

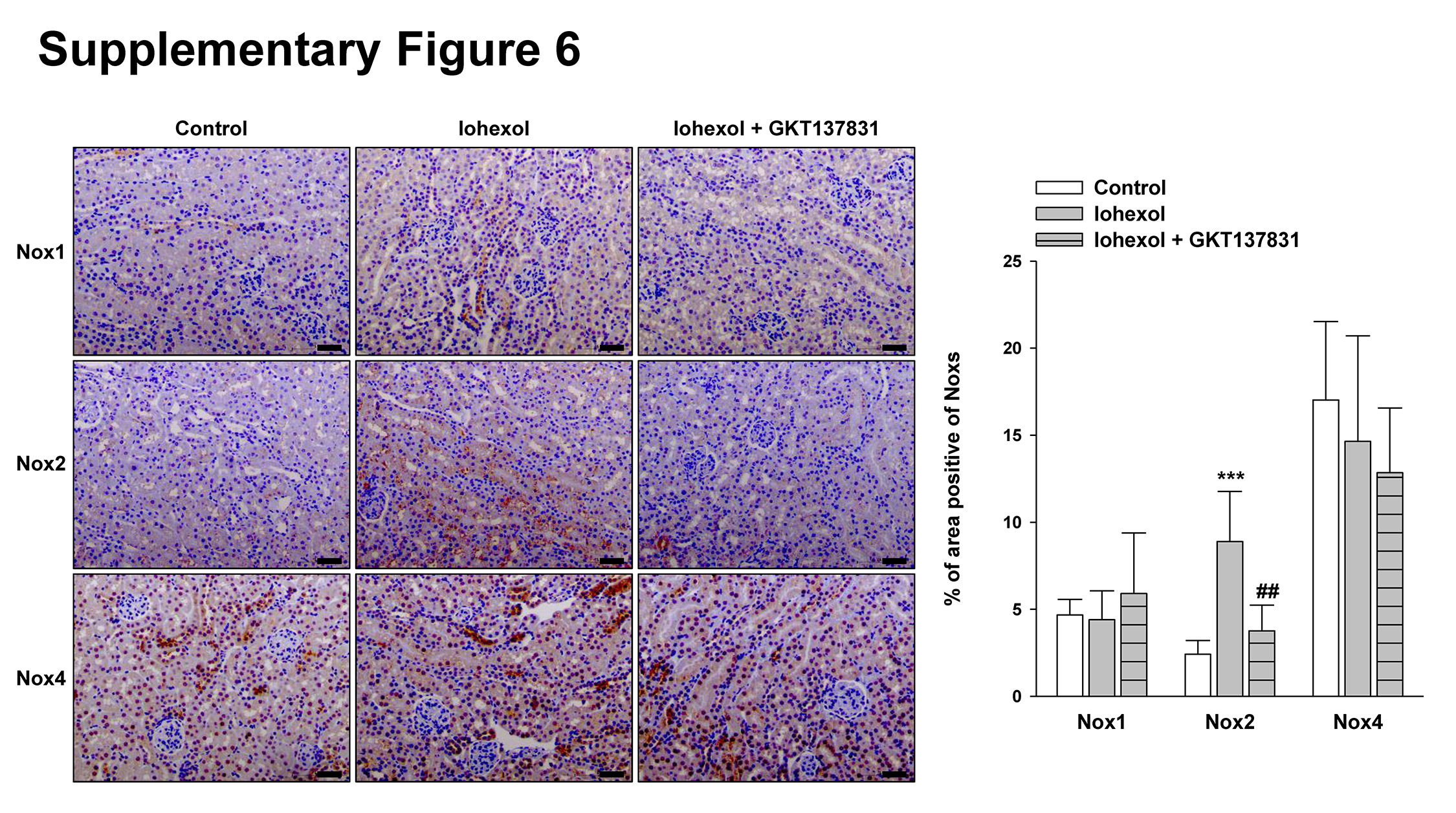

Supplement: S6 Fig — We performed immunohistochemistry (IHC) to determine the expression of Nox4 and other Noxs (Nox1 and Nox2) expression in vivo. The expression of Nox4 and Nox1 was not significantly different among control, iohexol and GKT pretreatment rats. Expression of Nox2 was significantly increased in iohexol rats and significantly decreased in GKT pretreatment rats. The data are the mean ± SD (n = 5). *p < 0.05 versus the control and ###p < 0.001 versus iohexol treatment only. (TIFF) [file pone.0191034.s006.tiff]

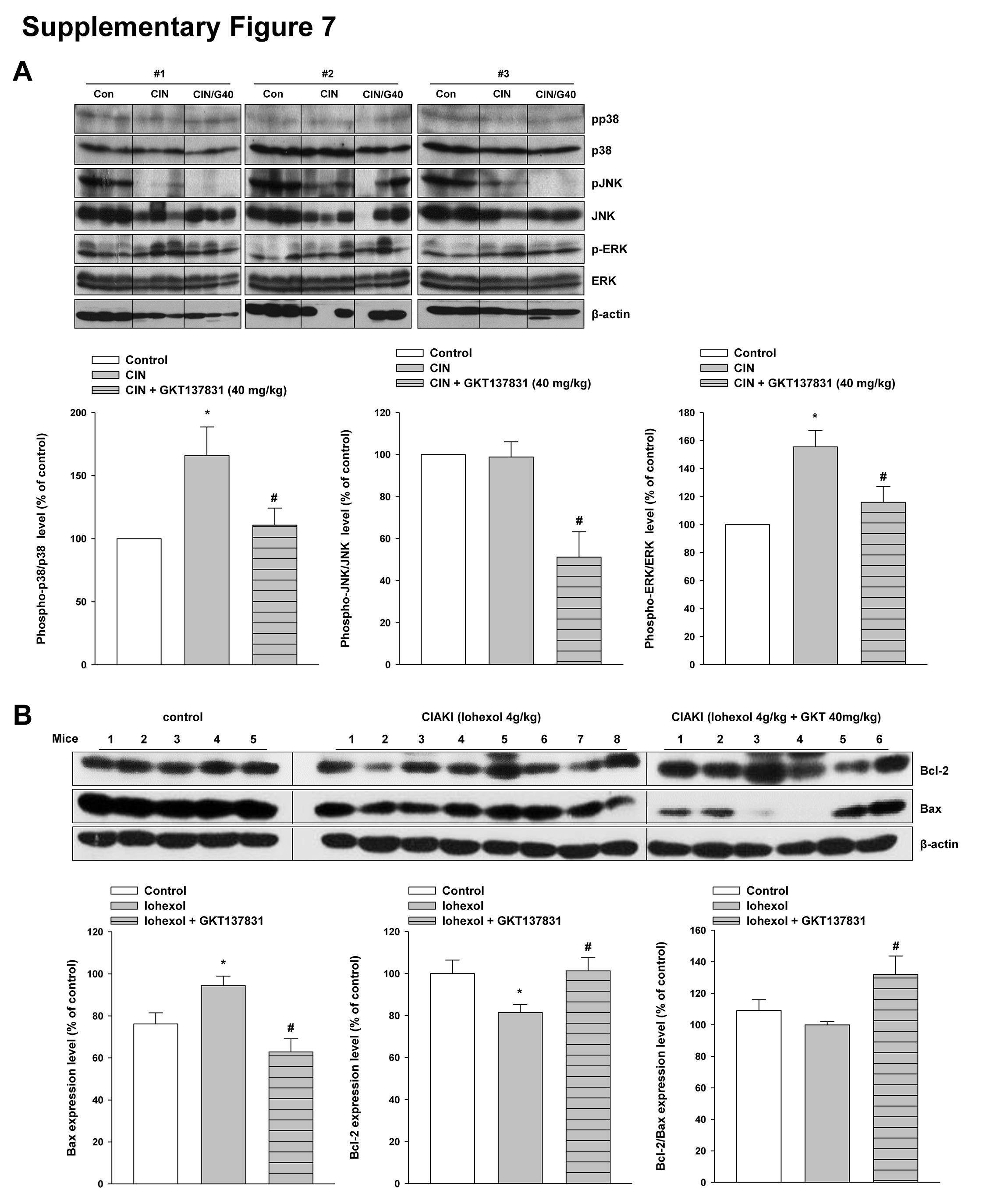

Supplement: S7 Fig — The protein levels of MAPKs (phospho-p38, p38, phospho-JNK, JNK, phosphor-ERK, ERK (A), Bax, and Bcl-2 (B) in kidney lysates from different groups were examined. Data are presented as the mean ± SD (n = 4–5). *p < 0.05 versus the control and #p < 0.05 versus Iohexol treatment only. (TIFF) [file pone.0191034.s007.tiff]
